# Analytical Model for Bond Behavior Prediction of CFRP-Concrete Joints with End Anchorage

**DOI:** 10.3390/polym13213684

**Published:** 2021-10-26

**Authors:** Kun Dong, Caiqun Zhong, Peng Li, Derun Du

**Affiliations:** Department of Civil Engineering, Ocean University of China, Qingdao 266100, China; dongkun@ouc.edu.cn (K.D.); zhongcaiqun@stu.ouc.edu.cn (C.Z.); ddrouc@ouc.edu.cn (D.D.)

**Keywords:** CFRP-to-concrete, end anchorage, analytical model, bond behavior, single shear test

## Abstract

Use of the end anchorages can significantly control the debonding of CFRP-to-concrete bond interface, and improve the bearing capacity of CFRP strengthened concrete member. An analytical model was presented in this paper to predict the bond behavior and debonding process of CFRP-concrete bonded joint with end anchorage. The calculation formulas of bond failure load and effective bond length for anchored CFRP-concrete joint are derived from the proposed analytical model. According to these models and formulas, the influence of different bond lengths on the mechanical behaviors during the debonding process was analyzed. Results show the load-slip curves of end anchored CFRP-concrete joints could be divided into three branches: elastic stage, stable stage, and enhancement stage. As the bond length increases, the plateau length in stable stage increases. Besides, the bond failure load decreased firstly to a lower limit and then increased with the increase of bond length. The effective bond length of CFRP-concrete joint with end anchorage was longer than that of the external bonded joint, and the value of effective bond length for end anchored joint shall be at least 7.2/*AB*, where the parameters *A* and *B* were related to the interfacial properties of bonded joint. Furthermore, a single shear test was carried out on the end anchored CFRP-concrete bonded joint with different bond lengths, to verify the consistency of the proposed model and formulas. The analytical result of load-slip response at the load end, as well as the strain distribution of CFRP material and the bond failure load, was compared with the experimental result. The comparisons showed that the analytical results had a good agreement with the experimental results.

## 1. Introduction

The reinforcement and repair of existing structures have become a critical problem due to the long-term service and overload damage. Due to the advantages of lightweight, high-strength, and good resistance to corrosion, carbon fiber-reinforced polymers (CFRP) has been widely used in the practice of retrofitting structures, such as fire-damaged concrete beam [1], earthquake-damaged concrete column [2], and shear wall [3]. The CFRP-strengthening technology has been accepted as an effective and efficient technology for enhancing the strength and stiffness of concrete structures [4,5,6]. The typical failure modes of CFRP strengthened sections usually appear as the debonding failure of bond interface between CFRP and concrete substrate [7]. Therefore, the strengthening efficiency will be greatly influenced by the failure mode. It has been suggested that additional end anchorage can be used to avoid debonding failure at the end of the bonding interface, which results in the increasing of the ultimate load [8]. More and more research has been conducted on additional anchored CFRP-strengthening technology.

In the existing research, a wide range of anchor types have been developed, typically including FRP anchor spike and steel anchor plate [9,10]. An anchor spike is rolled with a bundle of fibers, one end is embedded into the adhesive-filled hole, and the other end is fan-shaped glued onto the substrate. Ariyansyah et al. [11] indicated the anchor spike was the most effective anchorage method under the optimal spacing of CFRP anchor spike. Based on the experimental study, Alaa et al. [12] quantified the strength of CFRP anchors and analyzed the anchoring effect. However, the disadvantages of FRP anchor spike, large damage to components, complex construction, and long working hours requirement, are also obvious [13]. Steel anchor plate is used to cover the surface of CFRP composite, then is fixed on the substrate with steel bolts. Wu et al. [14,15] illustrated that the steel plate anchored CFRP strengthening method can significantly improve the bearing capacity of the concrete beam, yet the complex construction due to the multi-point anchorages was still inevitable. According to the tests conducted by Barris et al. [16], it is indicated that the effect of complete anchorage at the end sections can be achieved by applying sufficient torque to the bolt.

Considering the complex influence factors in actual experiments, the theoretical research is an alternative method to conveniently investigate the mechanical performance of FRP materials and strengthened members under different conditions, such as fatigue load [17], impact load [18], wet-dry cycling condition [19], etc. To characterize the debonding mechanism of the anchored CFRP-substrate interface, few theoretical models have been proposed. Based on the bilinear bond-slip constitutive model, Zhang et al. [20] and Sturm et al. [21] presented the load-slip relationship at the loading end and the distribution model of interfacial bonding performance for single-point and multi-point anchored FRP-substrate joints. Using the trilinear interface bond-slip model, Chen et al. [22] predicted the ultimate bearing capacity of the anchored bond interface, then presented an innovative design method for anchored CFRP-concrete joints. The above literature states that accurate analytical model is of great significance to predict the whole debonding process of CFRP-concrete bonded joints with end anchorage. However, there are still several problems worth noting. (i) The piecewise linear interface constitutive models were adopted in the above studies, which could not continuously reflect the nonlinear hardening and softening behavior of the bond interface. Different analytical solutions are needed to express the bond behavior for different interfacial stress states, which complicates the prediction process. (ii) Little discussion on the effective bonding length and the bond failure load for the end anchored joints were conducted in this literature.

The novelty of the proposed analytical model in this study is the prediction of the bond behavior and debonding process of CFRP-concrete bonded joint with end anchorage, based on a two-parameter exponential bond-slip curve. As mentioned above, the existing analytical models were established in the literature based on only bi-linear or tri-linear bond-slip relationship, and no analytical research was conducted due to the difficulties to get an analytical solution to the equilibrium differential equation related to a nonlinear exponential bond-slip curve. By the proposed analytical approach, the analytical solutions to predict bond stress along the interface and the slip, strain in the CFRP composite are reported here. Also, the calculation formulas of interfacial characteristics, including bond failure load and effective bond length, were deduced and the effect of different bond length was analyzed in detail. Finally, an experimental program was carried out on end anchored CFRP-concrete bonded joint with different bond lengths, and comparisons between the analytical solutions with the experimental results are presented.

## 2. Theoretical Background to the Analytical Model

### 2.1. Basic Assumptions

The development of the proposed analytical model in this paper aims to simulate the bond performance of CFRP-concrete interface with end anchorage, and the whole interfacial debonding process under external tensile load can be rapidly and accurately predicted with the presented model. In the derivation of the analytical model, several assumptions were taken to simplify the analysis:

1. The interface bears only the tangential bonding stress, and no normal stress yields;
2. CFRP and concrete are elastic, and the nonlinear mechanical characteristics including bond effect and failure only exist within the bond interface;
3. Regardless of the thickness of adhesive, the bond effect and failure of the bonding interface are reflected in the interfacial bond-slip model;
4. The CFRP stress is evenly distributed along the thickness direction, no considering the stress change in the width direction;
5. The anchor end is fully anchored, and the CFRP sheet at the anchor position does not have any slide during the whole loading process.

**Figure 1:**
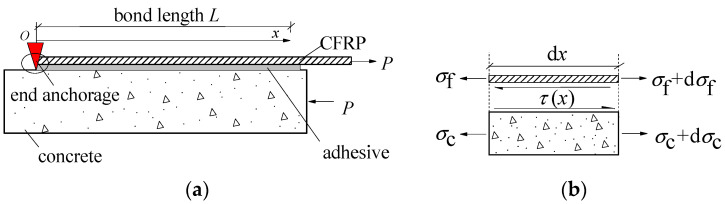
Single-lap shear test of CFRP-to-concrete bonded joints with end anchorage: (**a**) single-lap shear tests model; (**b**) Development of stress in CFRP–concrete joint.

The study on the debonding behavior of end anchored CFRP-concrete joint is based on single-lap shear tests, as schematically shown in Figure 1a. Note that the interface bond-slip model is entirely unrelated to the end anchorage, that is, the anchors do affect only the debonding behavior, but not the interfacial properties. Figure 1b shows the stresses developed in the CFRP and concrete substrate, considering the finite length d*x* of the bonded joint.

### 2.2. Differential Equilibrium Equations

According to the stress condition of finite length d*x* of the bonded joint in Figure 1b, the mechanical equilibrium equations can be established:

(1) $$\{\begin{array}{l} \frac{d\sigma_{f}}{dx}-\frac{\tau }{\tau_{f}}=0;\\ \sigma_{f}t_{f}b_{f}+\sigma_{c}t_{c}b_{c}=0. \end{array}$$

The relative slip between CFRP and concrete and the stress-displacement relationships of CFRP and concrete materials can be expressed as [7]:

(2) $$\{\begin{array}{l} s=u_{f}-u_{c};\\ \sigma_{f}=E_{f}\varepsilon_{f}=E_{f}\frac{du_{f}}{dx};\\ \sigma_{c}=E_{c}\varepsilon_{c}=E_{c}\frac{du_{c}}{dx}. \end{array}$$

Combining Equation (1) with Equation (2) can obtain:

(3) $$\frac{ds}{dx}=\left(1+\rho \right)\varepsilon_{f},$$

where $\rho =E_{f}t_{f}b_{f}/E_{c}t_{c}b_{c}$. Based on the calculation of the actual bonded joints in the existing single shear tests or strengthened members [16,19,23,24,25], the value of *ρ* was generally less than 0.01. Therefore, the parameter ρ is temporarily assumed to be zero in the subsequent derivation due to its little influence on the interfacial bond behavior.

The differential equilibrium equation about interfacial bond stress and slip can be obtained by the combination of Equations (1)–(3):

(4) $$\tau =E_{f}t_{f}\frac{d^{2}s}{dx^{2}}.$$

It has been indicated that the interfacial constitutive model is the key to analyze the bond behavior and debonding process of CFRP–concrete bonded joint. Dai et al. [26] proposed a two-parameter exponential bond-slip model as shown in Equation (5). The exponential model has two undetermined parameters and can be expressed as a smooth curve, which can succinctly and effectively reflect the nonlinear bond behavior between the CFRP and concrete substrate. Compared with the expressions derived from the bi-linear or tri-linear bond-slip models, the analytical expressions based on the exponential model are unified and continuous. A single expression without segmentation can reflect the behavior of the whole debonding process. At present, the exponential model has been widely used in the analytical analysis of the mechanical behavior of CFRP–concrete interface [27,28].

(5) $$\tau \left(s\right)=E_{f}t_{f}A^{2}B\left(1-e^{-Bs}\right)e^{-Bs},$$

where *A* and *B* are interfacial parameters, which can be obtained by shear tests on CFRP–concrete-bonded joint or simplified calculation method proposed by Dai et al. [7].

Introducing Equation (5) into Equation (4) yields the governing equilibrium differential equation of the debonding process of CFR–-concrete interface:

(6) $$\frac{d^{2}s}{dx^{2}}=A^{2}B\left(1-e^{-Bs}\right)e^{-Bs}.$$

### 2.3. Interfacial Slip and Stress Solution

In order to solve Equation (6), the boundary conditions of CFRP–concrete interfaces with end anchorage in Figure 1a should be given first. The CFRP strain at the anchor end (*x* = 0) is temporarily assumed as $\varepsilon_{0}$, which will be calculated later. In combination with Equation (3), the boundary conditions can be obtained:

(7) $$\{\begin{array}{l} x=0,\;\;\frac{ds}{dx}=\varepsilon_{0},\;s=0;\\ x=L,\frac{ds}{dx}=\frac{P}{E_{f}t_{f}b_{f}}. \end{array}$$

Integrate Equation (6), it can be obtained:

(8) $${\left(\frac{ds}{dx}\right)}^{2}=A^{2}{\left(1-e^{-Bs}\right)}^{2}+C_{1},$$

where *C*_1_ is an unterminated constant. Combined with the first term of boundary conditions in Equation (7), the expression of the slip strain is defined as:

(9) $$\frac{ds}{dx}=A\sqrt{{\left(1-e^{-Bs}\right)}^{2}+{\left(\frac{\varepsilon_{0}}{A}\right)}^{2}}\;.$$

Let $y=e^{-Bs\left(x\right)}$, $y_{0}^{2}=1+{\left(\frac{\varepsilon_{0}}{A}\right)}^{2}$, and substitute them into Equation (9)

(10) $$-\frac{1}{By}\frac{dy}{dx}=A\sqrt{{\left(1-y\right)}^{2}-\left(1-y_{0}^{2}\right)}.$$

Integrate Equation (10), we can get:

(11) $$-\frac{1}{y_{0}}\ln\left(\frac{\sqrt{y^{2}-2y+y_{0}^{2}}+y_{0}}{y}-\frac{1}{y_{0}}\right)=-ABx+C_{2},$$

where *C*_2_ is a constant determined from the boundary conditions of the debonding problem.

By simplifying Equation (11), the expression of variable *y* can be rewritten as [29]:

(12) $$y=\frac{2y_{0}^{3}e^{ABxy_{0}-C_{2}y_{0}}}{y_{0}^{2}e^{2ABxy_{0}-2C_{2}y_{0}}+2y_{0}e^{ABxy_{0}-C_{2}y_{0}}+\left(1-y_{0}^{2}\right)}.$$

Imposing the boundary condition *y* (0) = 1 in Equation (12), constant *C*_2_ is calculated as:

(13) $$C_{2}=\frac{1}{y_{0}}\ln\frac{y_{0}}{y_{0}^{2}-1+y_{0}\sqrt{y_{0}^{2}-1}}.$$

Let $\varphi =\frac{\varepsilon_{0}}{A}$, then replace $y_{0}^{2}=1+\varphi^{2}$ and constant *C*_2_ back to Equation (12) yields:

(14) $$y=\frac{1+\varphi^{2}}{1+\varphi^{2}\cos h(ABx\sqrt{1+\varphi^{2}})+\varphi \sqrt{1+\varphi^{2}}\sin h(ABx\sqrt{1+\varphi^{2}})}.$$

Finally, combined with $y=e^{-Bs\left(x\right)}$, the analytical formula of interfacial slip can be obtained:

(15) $$s\left(x\right)=\frac{1}{B}\ln\frac{1+\varphi^{2}\cos h(ABx\sqrt{1+\varphi^{2}})+\varphi \sqrt{1+\varphi^{2}}\sin h(ABx\sqrt{1+\varphi^{2}})}{1+\varphi^{2}}.$$

The distribution formula of the CFRP strains can be obtained by combining Equations (3) and (15):

(16) $$\varepsilon_{f}\left(x\right)=A\varphi +\frac{A\varphi \left[cosh\left(ABx\sqrt{1+\varphi^{2}}\right)-1\right]}{1+\varphi^{2}cosh\left(ABx\sqrt{1+\varphi^{2}}\right)+\varphi \sqrt{1+\varphi^{2}}sinh\left(ABx\sqrt{1+\varphi^{2}}\right)}\;.$$

At the same time, the distribution formula of longitudinal CFRP stress can be obtained in combination with Equation (2):

(17) $$\sigma_{f}\left(x\right)=E_{f}A\varphi +\frac{E_{f}A\varphi \left[cosh\left(ABx\sqrt{1+\varphi^{2}}\right)-1\right]}{1+\varphi^{2}cosh\left(ABx\sqrt{1+\varphi^{2}}\right)+\varphi \sqrt{1+\varphi^{2}}sinh\left(ABx\sqrt{1+\varphi^{2}}\right)}.$$

The distribution formula of the bond stress can be obtained by Equations (1) and (17):

(18) $$\tau_{f}\left(x\right)=E_{f}t_{f}\frac{A^{2}B\varphi \left(1+\varphi^{2}\right)\left[\sqrt{1+\varphi^{2}}\sin h(ABx\sqrt{1+\varphi^{2}})+\cos h(ABx\sqrt{1+\varphi^{2}})-1\right]}{{\left[1+\varphi^{2}\cos h(ABx\sqrt{1+\varphi^{2}})+\varphi \sqrt{1+\varphi^{2}}\sin h(ABx\sqrt{1+\varphi^{2}})\right]}^{2}}.$$

### 2.4. External Load—Slip Response

Substituting the second term in Equation (7) into Equations (3) and (16), we can get:

(19) $$P=E_{f}b_{f}t_{f}A\varphi +\frac{E_{f}b_{f}t_{f}A\varphi \left[\cos h\left(ABL\sqrt{1+\varphi^{2}}\right)-1\right]}{1+\varphi^{2}\cos h\left(ABL\sqrt{1+\varphi^{2}}\right)+\varphi \sqrt{1+\varphi^{2}}\sin h\left(ABL\sqrt{1+\varphi^{2}}\right)}.$$

Combined with $\varphi =\varepsilon_{0}/A$, Equation (19) gives the one-to-one correspondence between the external load *P* and the CFRP strain $\varepsilon_{0}$ at the anchor end. At this time, the only CFRP strain $\varepsilon_{0}$ at the anchor end can be obtained under a given load, and other physical expressions can be determined according to Equation (15) to Equation (19).

By substituting *x* = *L* into Equation (15), the slip at the load end is given as follows:

(20) $$s\left(L\right)=\frac{1}{B}ln\frac{1+\varphi^{2}\cos h\left(ABL\sqrt{1+\varphi^{2}}\right)+\varphi \sqrt{1+\varphi^{2}}\sin h\left(ABL\sqrt{1+\varphi^{2}}\right)}{1+\varphi^{2}}\;.$$

**Figure 2:**
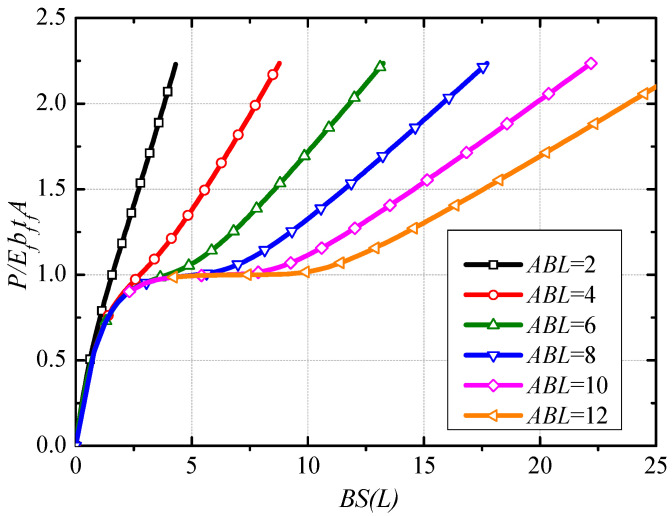
Normalized Load-slip response under different bond length.

The load-slip response curve at the load end can be obtained by the simultaneous equations of Equations (19) and (20). Figure 2 shows the normalized load-slip curves with different normalized bond length *ABL*.

From Figure 2, three branches can be defined as: (i) Elastic stage, the tensile load has a nearly linear development with the increase of slip at the load end; (ii) Stable stage, when normalized bond length is greater than a certain value, the plateau stage occurs due to the interface debonding from the load end to anchor end. The tensile load corresponding to the plateau stage approximately equals to $E_{f}b_{f}t_{f}A$. As the increase of normalized bond length, the length of the plateau stage increases; (iii) Enhancement stage, this branch exists because of the presence of end anchorage, i.e., most of the tensile load is borne by the anchors in the stage. With the continued increase of slip, the slope of the curve tends to the axial tensile stiffness of CFRP.

## 3. Interface Characteristics

### 3.1. Bond Failure Load

According to the analytical model deduced above, it can be obtained that the external load *P* of the whole bonding interface can be divided into two parts, the anchor force *P_a_* and the bond force *P_b_*. The mathematical expression is as follows:

(21) $$P=P_{a}+P_{b},$$

where $P_{b}=\int_{0}^{L}\tau \left(x\right)b_{f}\mathrm{dx}$., i.e., the integral value of bond stress along the whole bond interface. According to the static equilibrium equation of the anchored point and Equation (17), the anchor force *P_a_* is expressed as:

(22) $$P_{a}=b_{f}t_{f}\sigma_{f}\left(0\right)=E_{f}b_{f}t_{f}A\varphi ,$$

By combining Equations (19), (21) and (22), the bond force *P_b_* can be obtained as follows:

(23) $$P_{b}=\frac{E_{f}b_{f}t_{f}A\varphi \left[\cos h\xi_{0}-1\right]}{1+\varphi^{2}\cos h\xi_{0}+\varphi \sqrt{1+\varphi^{2}}\sin h\xi_{0}}\;,$$

where $\xi =ABL\sqrt{1+\varphi^{2}}$.

**Figure 3:**
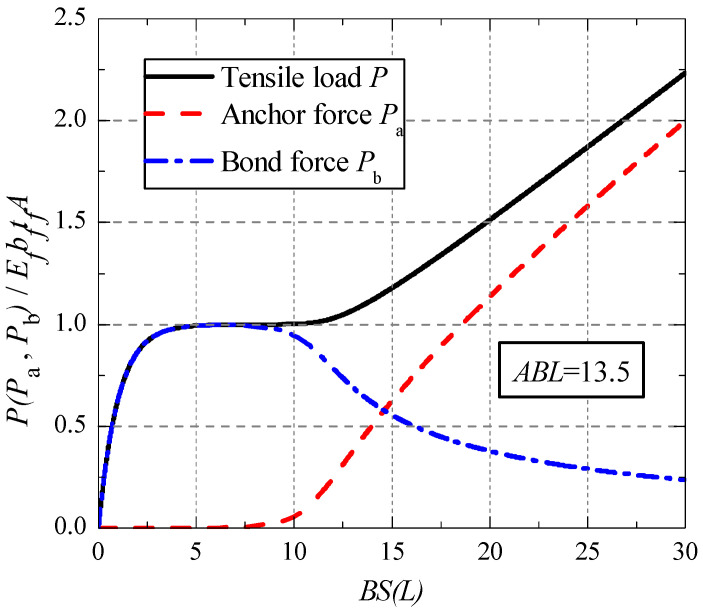
Relationship between *P* (*P_a_*, *P_b_*) and slip at load end.

Take a bonded joint with a normalized bond length of 13.5 for example, the relationships of *P*, *P_a_* and *P_b_* with the slip s are shown in Figure 3. It can be seen that, for this bonded joint, the anchor force *P_a_* begins to work after the bond force *P_b_* reaches the maximum, at that time the debonding behavior begins. In this situation, the bond failure load *P_d_* can be defined as the tensile load corresponding to the maximum bond force.

By deriving the extreme value of Equation (23), the expression of the bond failure load for a given bonded joint can be obtained as follows:

(24) $$P_{d}=E_{f}b_{f}t_{f}A\varphi_{0}+\frac{E_{f}b_{f}t_{f}A\varphi_{0}\left[cosh\xi_{0}-1\right]}{1+\varphi_{0}^{2}cosh\xi_{0}+\varphi_{0}\sqrt{1+\varphi_{0}^{2}}\;sinh\xi_{0}}.$$

The $\varphi_{0}$ in Equation (24) can be obtained by the following formula:

(25) $$\left(\varphi_{0}^{2}\cos h\xi_{0}+\frac{\varphi_{0}^{3}}{\sqrt{1+\varphi_{0}^{2}}}\sin h\xi_{0}-ABL\varphi_{0}^{3}-1\right)\left(\cos h\xi_{0}-1\right)=\varphi_{0}^{2}\xi_{0}\sin h\xi_{0\;}.$$

**Figure 4:**
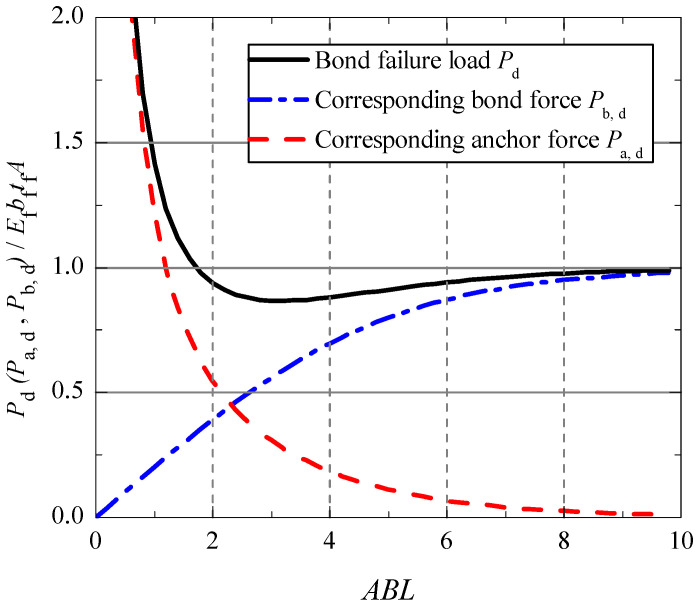
Relationship between *P_d_* (*P_a_*,_*d*_, *P_b_*,_*d*_) and bond length.

The corresponding anchor force *P_a_*_,*d*_ and bond force *P_b_*_,*d*_ can be obtained by introducing Equation (25) into Equations (22) and (23). Figure 4 shows the evolution curves of *P_d_*, *P_a_*_,*d*_ and *P_b_*_,*d*_ along with the change of bond length.

From Figure 4, it can be found that the bond failure load decreased firstly to a lower limit at *ABL* = 3 and then increased with the increase of bond length. However, the force *P_a_*_,*d*_ is a monotonous decreasing function, and the force *P_b_*_,*d*_ is a monotonous increasing function with the bond length. It is indicated that the end anchorage did not play an important effect before the bond failure for a long bonded joint. For a short bonded joint (*ABL* smaller than 2), the anchor force took more than 50 percent of the total tensile load when the bond failure load was reached.

### 3.2. Effective Bond Length

**Figure 5:**
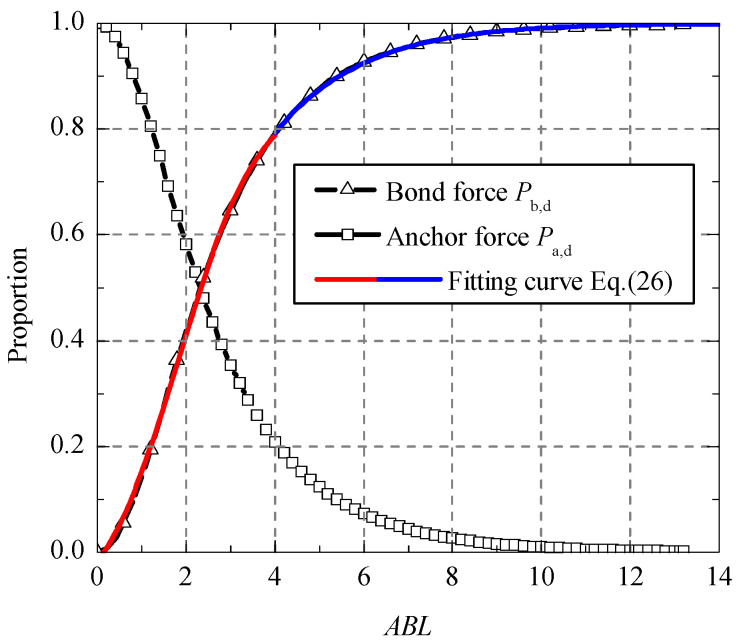
Load proportion curves.

According to the relationship of the characteristic loads and bond length in Figure 4, the proportion of bond force *P_b_*_,*d*_ in the bond failure load *P_d_* is defined as *α*, which can be drawn in Figure 5. By fitting analysis, the simple expression of ratio *α* can be presented as:

(26) $$\alpha =\{\begin{array}{l} 0.37-0.51tanh\left(-0.54ABL+1\right),\;\;\;0\leq ABL\leq 4;\\ -tanh\left(-0.27ABL\right),\;\;\;ABL>4. \end{array}$$

For the externally bonded CFRP strengthened concrete joint, when the bond force *P_b_*_,d_ accounts for more than 96% of the external failure load *P_d_*, the corresponding bond length is defined as the effective bond length *L*_eff_ [7]. The same definition method is used here to determine the effective bond length of end anchored CFRP-concrete joint. By solving the inverse function of the second part of Equation (26), the calculation formula of *L*_eff_ can be obtained as:

(27) $$L_{\mathrm{eff}}=\frac{1.85}{AB}\ln\frac{1+\alpha }{1-\alpha },\;\alpha \geq 0.96.$$

By considering the existance of the free end slip, Dong et al. [30] gives the expression of ratio α for the externally bonded CFRP–concrete interface without end anchorage:

(28) $$\alpha =\{\begin{array}{l} 1.761tanh\left(0.142ABL\right)\;,\;0\leq ABL\leq 2;\\ \tan h(0.332ABL-0.132)\;,ABL>2. \end{array}$$

Similarly, by finding the inverse function of the second part of Equation (28), the calculation formula of the effective bond length for the externally bonded joint can be obtained as follows:

(29) $$L_{\mathrm{eff}}=\frac{1.5}{AB}\ln\frac{1+\alpha }{1-\alpha }+\;\frac{0.4}{AB},\;\;\alpha \geq 0.96.$$

**Figure 6:**
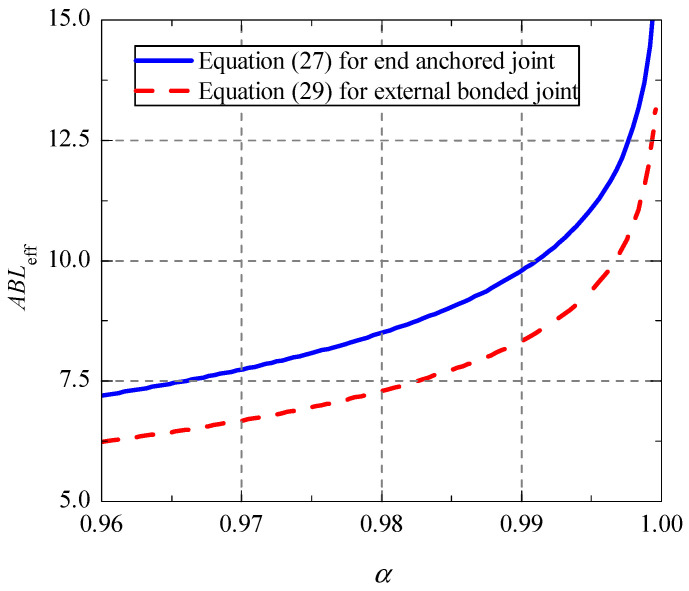
Relationship between effective bond length and ratio α.

As can be seen in Figure 6, compared with the external bonded joint, the CFRP–concrete joint with end anchorage requires for the larger effective bond length at same ratio α and the difference between them is about 1/*AB*. In addition, the effective bond length for the joint with end anchorage shall be at least 7.2/*AB*.

## 4. Validation of the Presented Analytical Model

### 4.1. Experimental Program

**Figure 7:**
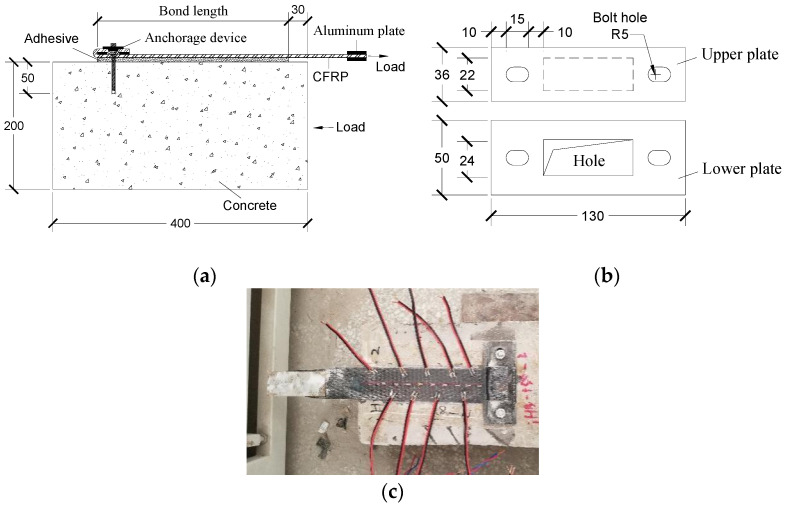
CFRP–concrete bonded joint with self-locked end anchorage: (**a**) Single-shear test joint with end anchorage; (**b**) Size drawing of steel plates; (**c**) Layout of strain gauges.

To validate the accuracy of the above analytical models, a single shear experimental program on CFRP–concrete joints with mechanical end anchorages were carried out to collect the test data for comparison. First, two externally bonded CFRP–concrete joints were tested to determine the two-parameter exponential bond-slip model. Then, take ratio *α* = 0.995, the effective bond length for end anchored joints was 123 mm. Therefore, three different bond lengths were applied in the test, that was 100 mm (*L* < *L*_eff_), 150 mm (*L* > *L*_eff_) and 200 mm (*L* > *L*_eff_), respectively. Each concrete substrate was 200 mm wide, 200 mm thick and 400 mm long. Detail reinforcement and anchorage information of the tested specimens are shown in Table 1 and Figure 7.

As can be seen in Figure 7a,b, CFRP was bonded to the concrete surface adopted epoxy resin adhesive. It should be noted that a new self-locked end anchorage device, consisted of two steel plates with thickness of 5 mm, was used to fasten the free end of CFRP in this experiment. And both ends of the plate were fixed with 8 mm diameter and 50 mm depth high-strength bolts. Besides, the aluminum plate was set at the load end to improve the friction. As shown in the Figure 7c, strain gauges were attached on the surface of CFRP with a space of 20 mm along the longitudinal direction. According to ASTM C39 [31], the cylindrical compressive strength and elastic modulus of concrete was measured as 37 MPa and 33 GPa, respectively. For CFRP composites, the tensile strength was 2870 MPa and the elastic modulus was 220 GPa, according to ASTM D3039 [32].

**Figure 8:**
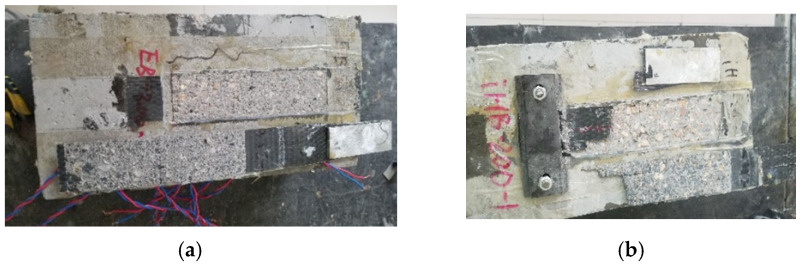
Failure of specimens: (**a**) EB-200-0; (**b**) SLHB-200-15.

Table 2 and Figure 8 shows two kinds of the representative failure modes in the test. The traditional externally bonded specimens failed with typical debonding, while the failure form of end anchored specimens was the fracture of CFRP composites. For end anchored specimens, the debonding process in the early stage was consistent with the external bonded specimens, that is, the relative slip *s* at the loading end increased with the increase of external load until debonding began. During this period, the interfacial bond stress played a main role. After interface debonding, most tensile load was borne by the anchorage device. With the existing of end anchorage, the specimens can successfully bear the increasing load until the load reaches a certain value of CFRP fracture. Unlike the sudden debonding of CFRP in the test on externally bonded specimens, sudden fracture of CFRP occurred at the end of the test on end anchored specimens.

According to the average values of ultimate loads and slips in each group (see Table 2), the average ultimate load and corresponding slip of anchored specimens were much larger than those of externally bonded specimens. Compared with the EB-200 specimens, the ultimate load and slip of EA-200 specimens were improved by about 57% and 170%, respectively.

### 4.2. Results of Comparisons

#### 4.2.1. Load-Slip Response

**Figure 9:**
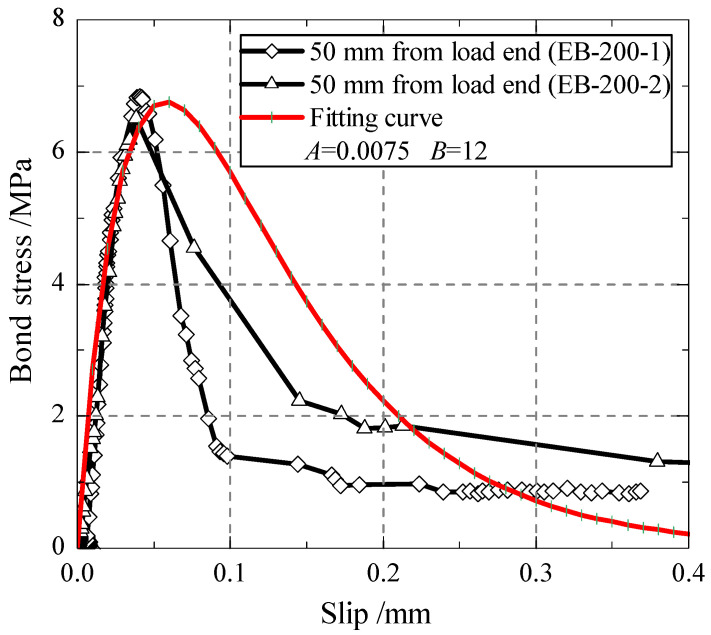
Bond-slip constitutive model.

Based on the CFRP strain distribution measured in the test on externally bonded specimens, the interfacial slip and bonded stress were gained according to the integral conversion method given by He et al. [33]. By fitting the experimental bond stress-slip curves, the theoretical bond-slip model of the CFRP–concrete interface is obtained in the form of Equation (5), where the parameters *A* = 0.0075 and *B* = 12 (seen in Figure 9). The fitting curve can represent well the interfacial constitutive of CFRP–concrete specimens, especially in the ascending portion of the curve.

**Figure 10:**
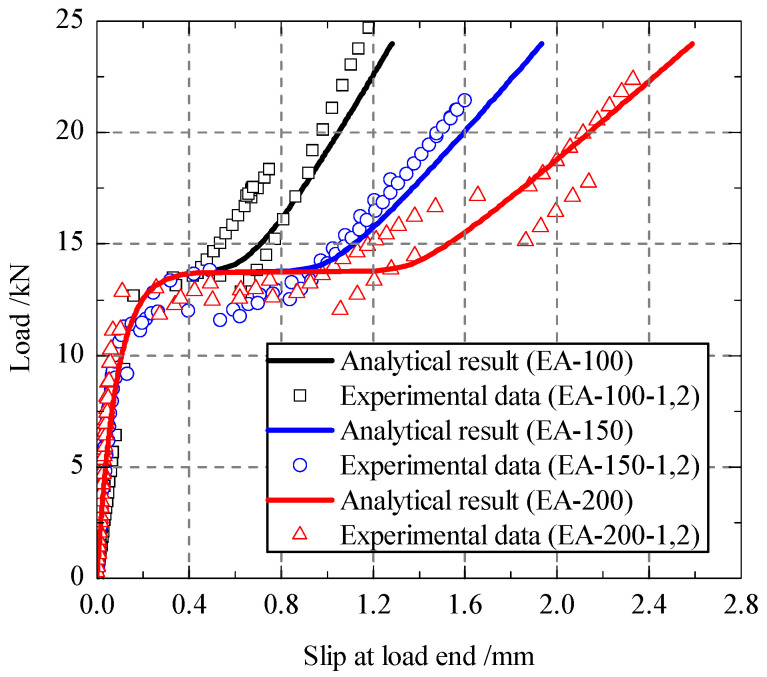
Load-slip response.

In terms of the fitting interfacial bond-slip model and the analytical load-slip curves were deduced according to Equations (19) and (20). As shown in Figure 10, the analytical results were compared with the experimental results. It is obvious that the analytical load-slip curves were in good agreement with the experimental results. However, the experimental ultimate loads were little lower than the analytical ultimate load, which may be attributed to the uneven stress of CFRP material during the test.

#### 4.2.2. Strain Distribution

**Figure 11:**
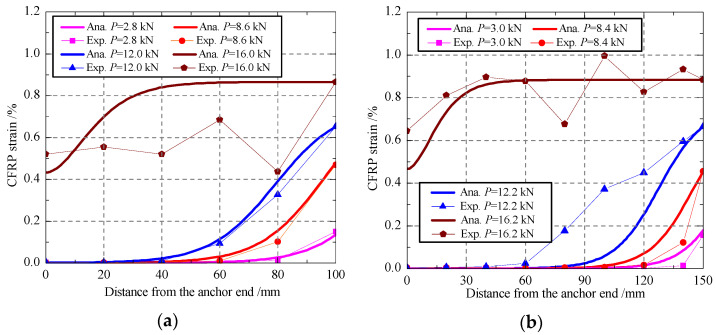

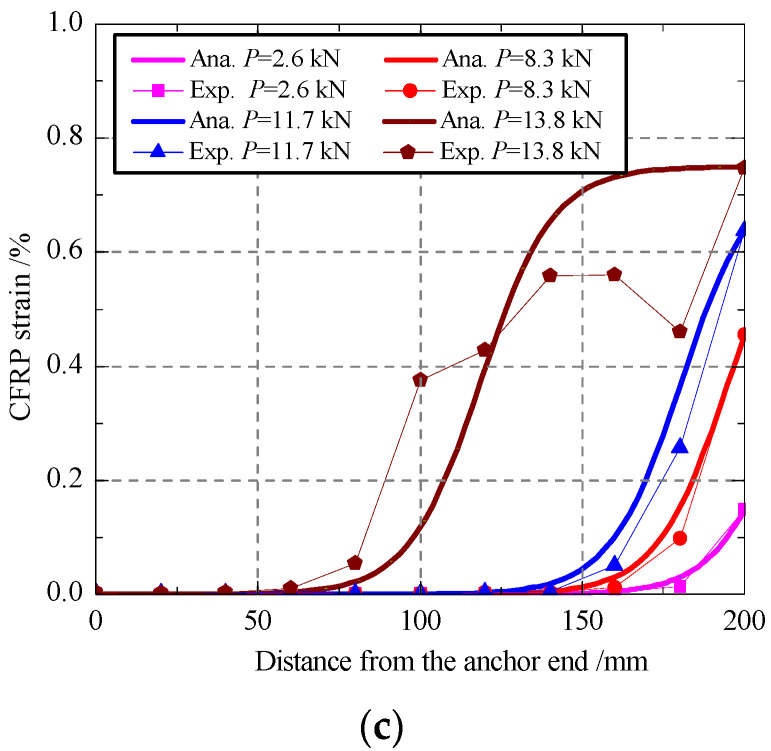
Distributions of the CFRP strains: (**a**) EA-100-1; (**b**) EA-150-1; (**c**)EA-200-1.

According to the presented analytical model in Section 2, the distributions of CFRP strain under different loads were calculated with Equation (16) and then compared with the measured data in the experimental program, as shown in Figure 11. Under an external load lower than the bond failure load, the analytical results are in a good agreement with the measured data. For a small load, there is generally no strain distribution in the vicinity of the anchor end, but the strain increases with the increasing of the distance from the anchor end. When the external load increases gradually, the CFRP strain near the loading end increases quickly until the bond failure load is reached. After the bond failure load, the strains do not change in a certain distance from the loading end to the anchor end, which indicates that the debonding failure happened and developed towards to the anchor end. At that stage, the strains near the anchor end grow very fast, and the strain distribution curve became nearly a straight line until the CFRP fracture. For the bond behavior in the stage after bond failure, the predictions of the proposed analytical have some deviations with the experimental results, which may be due to the effect of uneven high strain state and the dynamic process of interface debonding on the stain measurement. Generally, the presented model has a good prediction on the whole bond and debonding process of end anchored CFRP–concrete joint.

#### 4.2.3. Bond Failure Load and Slip

Table 3 shows the comparisons of the bond failure load *P_d_* and the corresponding slip *s_d_* between experimental and the analytical results. Noted that the experimental data are the average of the two specimens with the same bond length.

No obvious plateau stage for the joint with a bond length of 100 mm. Therefore, it is hard to get the bond failure load from the experimental curves. For the joints with a bond length of 150 mm or 200 mm, the bond failure load can be recognized as the peak load in the plateau stage, because the bond failure could cause a sudden drop of the load during the test. In Table 3, with the increasing of bond length, the analytical prediction *P_d_* obtained by Equation (24) increases, i.e., 13.57 kN for 100 mm, and 13.75 kN for 150 mm. When the bond length exceeds the effective bond length (123 mm), the failure load *P_d_* and the slip *s_d_* no longer increases with the bond length. According to the comparisons, the ratio of analytical prediction to experimental result is between 1.02–1.14, which indicates that the presented model in this paper has a satisfactory predicting accuracy.

## 5. Conclusions

A nonlinear analytical approach was presented in the paper for predicting the whole bond and debonding process of end anchored CFRP–concrete bonded joints. A two-parameter exponential bond-slip model was assumed for the bond interface. According to the previous analytical derivation and discussion, the main conclusions can be drawn as follows:

1. The analytical solutions provide a series of expressions for the, including interfacial stress, CFRP strain, load-slip response, bond failure load and effective bond length, which are helpful for fully understanding the whole bond and debonding process of CFRP–concrete interfaces with end anchorage;
2. A single-lap shear test has been conducted to validate the accuracy of the above analytical solutions. Based on two-parameter exponential bond-slip model, the analytical predictions had a good agreement with the experimental results, in the aspects of load-slip response, CFRP strain distribution and bond failure load. It is indicated that the presented analytical approach can predict well the bond performance of end anchored CFRP–concrete joints with different bond lengths.
3. The load-slip behavior of end anchored CFRP–concrete joints could be divided into three branches: (i) Elastic stage, the tensile load has a nearly linear development; (ii) Stable stage, the plateau stage occurs approximately at the value of $E_{f}b_{f}t_{f}A$. As the increase of normalized bond length, the length of the plateau stage increases; (iii) Enhancement stage, the anchors play a major role in the stage. With the continued increase of slip, the slope of the curve tends to the axial tensile stiffness of CFRP.
4. With the increase of bond length, bond failure load decreased firstly to a lower limit at *ABL* = 3 and then gradually increased to the ultimate value of $E_{f}b_{f}t_{f}A$.
5. The CFRP–concrete joints with end anchorage need a larger effective bond length than the external bonded joints. The effective bond length for the end anchored joints shall be at least 7.2/*AB*.

The analytical model proposed in this paper can help engineers to understand the anchoring effect and failure mode of CFRP–concrete joints with end anchorage. It also provides engineers with design suggestions for choosing the bond length and calculating the bond failure load of end anchored joints.

## Figures and Tables

**Table 1:** Information of specimens.

Specimen Code	Bond Length (mm)	Bond Width (mm)	CFRP Thickness (mm)	Anchor Form
EB-200-1,2	200	50	0.167	EB
EA-100-1,2	100			EA
EA-150-1,2	150			EA
EA-200-1,2	200			EA

Note: In the specimen code, ‘EB’ represents ‘externally bonded’; ‘EA’ represents ‘end anchored’; ‘200′ represents the joint has a bond length of 200 mm; ‘1,2′ represents the 1st and 2nd specimen with the same design in one group.

**Table 2:** Failure modes.

Specimen Code	Ultimate Load *P*_u_/KN	Ultimate Slip *s*_u_/mm	Failure Mode
EB-200-1,2	12.96	0.8355	Debonding failure of Interface
EA-100-1,2	21.37	0.9466	Fracture failure of CFRP
EA-150-1,2	20.54	1.4758	Fracture failure of CFRP
EA-200-1,2	20.29	2.2557	Fracture failure of CFRP

**Table 3:** The *P***_d_** and *s*_d_ from experimental and analytical results.

Specimens	Bond Failure Load *P_d_*/kN			Bond Failure Slip *s_d_*/mm		
	Exp.	Ana.	Ana./Exp.	Exp.	Ana.	Ana./Exp.
EA-100-1,2	--	13.57	--	--	0.3477	--
EA-150-1,2	12.98	13.75	1.06	0.4460	0.5088	1.14
EA-200-1,2	12.83	13.75	1.07	0.5002	0.5080	1.02

## Data Availability

All data included in this study are available upon request by contact with the corresponding author.

## Nomenclature

$b_{c}$	width of concrete
$b_{f}$	width of CFRP composite
$E_{c}$	elastic modulus of concrete
$E_{f}$	elastic modulus of CFRP composite
L	bond length of CFRP-concrete joint
$L_{eff}$	effective bond length
P	external tensile load at the load end
$P_{a}$	anchor force at the anchor end
$P_{b}$	bond force
$P_{d}$	bond failure load
$P_{a,d}$	anchoring force corresponding to the bond failure load
$P_{b,d}$	bond force corresponding to the bond failure load
*s*	slip or relative displacement between the CFRP and the concrete
$s_{d}$	slip corresponding to the bond failure load
$t_{c}$	thickness of concrete
$t_{f}$	thickness of CFRP composite
$u_{c}$	displacement of the concrete
$u_{f}$	displacement of CFRP composite
α	ratio of anchor load to external load
$\varepsilon_{0}$	strain in the CFRP composite at the anchor end
$\varepsilon_{c}$	strain in concrete
$\varepsilon_{f}$	strain in the CFRP composite
τ	interfacial bond stress
$\tau_{f}$	bond strength of CFRP-concrete Joint
$\sigma_{c}$	stress of the concrete
$\sigma_{f}$	stress of the CFRP composite
